# Biofilm containing the *Thymus serpyllum* essential oil for rice and cherry tomato conservation

**DOI:** 10.3389/fpls.2024.1362569

**Published:** 2024-03-08

**Authors:** Josefa Roselló, Juan Antonio Llorens-Molina, Silvina Larran, Francisca Sempere-Ferre, M. Pilar Santamarina

**Affiliations:** ^1^ Departamento de Ecosistemas Agroforestales, Universitat Politècnica de València, València, Spain; ^2^ Instituto Agroforestal Mediterráneo, Universitat Politècnica de València, València, Spain; ^3^ Centro de Investigaciones de Fitopatología, Facultad de Ciencias Agrarias y Forestales, Universidad Nacional de La Plata, Buenos Aires, Argentina; ^4^ Departamento de Estadística e Investigación Operativa Aplicadas y Calidad, Universitat Politècnica de València, València, Spain

**Keywords:** biofilm, antifungal, *Thymus*, essential oils, post-harvest, cherry tomato, rice, conservation

## Abstract

**Introduction:**

Fungal pathogens cause major yield losses in agriculture and reduce food quality and production worldwide.

**Purpose:**

To evaluate new safer alternatives to chemicals for disease management and preserve the shelf life of food, this research was conducted to: determine the chemical composition of the essential oils (EOs) of *Thymus serpyllum* and *Thymus piperella* chemotypes 1 and 2; investigate the antifungal potential of EOs *in vitro* against: *Alternaria alternata*, *Bipolaris spicifera*, *Curvularia hawaiiensis*, *Fusarium oxysporum* f. sp. *lycopersici*, *Penicillium italicum*, *Botryotinia fuckeliana*; evaluate a natural *T. serpyllum* extract biofilm to conserve rice grain and cherry tomatoes.

**Method:**

EOs were analyzed by GC-MS+GC-FID. EOs’ antifungal activity was evaluated by dissolving *Thymus* extracts in PDA. Petri dishes were inoculated with disks of each fungus and incubated at 25°C for 7 days.

**Results:**

The *T. serpyllum* EO displayed the best Mycelial Growth Inhibition. The antifungal effect of the *T. serpyllum* EO biofilm was evaluated on rice caryopsis. Disinfected grains were dipped in a conidial suspension of each fungus and sprayed with EO (300 and 600 μg/mL) prepared in Tween 20. Grains were stored. The percentage of infected grains was recorded for 30 days. The *T. serpyllum* EO effect on cherry tomato conservation was evaluated *in vivo*. Wounded fruit were immersed in the *T. serpyllum* EO (300 and 400 μg/mL) and inoculated with *Fusarium oxysporum* f. sp. *lycopersici*. Fruit were evaluated for 7 and 14 days. Chemical profiles thymol/carvacrol for *T. serpyllum*, carvacrol for *T. piperella* Tp1 and thymol for *T. piperella* Tp2 were defined. The three evaluated EOs reduced all the studied phytopathogens’ fungal growth. The *T. serpyllum* biofilm was effective with rice storage and against *Fusarium oxysporum* f. sp. *lycopersici* for extending the shelf life of tomatoes in warehouses and storing postharvest cherry tomatoes.

**Conclusion:**

We suggest applying these EOs as biofilms for safe food conservation to replace synthetic products.

## 1 Introduction

It is widely known worldwide that global food demand is increasing as the world´s population grows, which is projected to reach 8.6 billion people in 2030 and 9.8 billion in 2050 (United Nations, 2017). On the one hand, cereal grains, such as rice, wheat and maize, are major staple foods grown in most of the world, and supply a major proportion of people’s energy and nutritional needs. In particular, rice is staple food for people all around the world, particularly in Asia, Latin America and parts of Africa. In 2020, cereals were the main crops to be globally produced with slightly less than one third of total crops (FAO, 2022). The foods that are predominantly carbohydrate are important because they form the basis of most diets, especially for the poorest people in the developing world. In developing countries, these foods generally supply 70%, or more, of the population’s energy intake. On the other hand, tomato (*Solanum lycopersicum* L.) contributes considerably to human nutrition. It is a source of mainly carbs, vitamin C, fiber, vitamin k, among other components (United States Department of Agriculture (USDA), 2018). This crop is one of the most important horticultural crops in the world. According to FAOSTAT (2022), Spain ranked seventh as a world producer in 2021 with 4754.380 million metric tons, where tomato was grown on 56.11 ha. In Argentina, this crop is cultivated under greenhouse and field conditions. Argentina is another of the world’s largest producers, where tomato growing annually occupies around 15000 ha (Malbrán et al., 2020).

It is important to consider that hunger is still on the rise with almost 770 million people undernourished in 2021, which is an increase of 150 million since 2019. Agriculture and food production systems affect food availability and affordability, as well as diet quality and diversity (Food and Agriculture Organization of the United Nations (FAO), 2016). In line with all this, several scientists have been working hard in recent years to maximize food production and to reduce pre- and postharvest crop losses. The world’s production of primary crops has increased 52% between 2000 and 2020, which is mostly attributable to better crop management technology, among other factors (FAO, 2022).

Fungal pathogens are biotic adversities that cause major yield losses in agriculture, and reduce food quality and production worldwide (Almeida et al., 2019). Losses of between 35-55% caused by postharvest diseases of fruit and vegetables have been reported, with more significant values in developing countries (Verdeguer et al., 2020). Another important issue to highlight is that fungal proliferation in stored food and cereal not only causes significant harvest quality and yield losses (rancid odors and flavors), but some fungal species also produce secondary potentially toxins like aflatoxins and fumonosins, which pose human health problems (Soliman and Badeaa, 2002). Of the 17 Sustainable Development Goals (SDGs) agreed by world leaders in 2015, some particularly relevant ones are zero hunger and responsible consumption and production by considering not only higher production levels, but also reducing food losses throughout production and supply chains, including postharvest losses and better environmental care (Naciones Unidas, 2023; United Nations, 2023).

In recent years, society has tended to reduce chemical products like synthetic fungicides to control the phytopathogenic fungi of crops and postharvest diseases, which has led researchers to evaluate and develop new safer alternatives for the environment, and also for human and animal health. Aromatic plants and their essential oils (EOs) have been widely known since ancient times. They are used in foods to enhance flavor and organoleptic properties, and in the pharmaceutical industry for their beneficial effects on health. Nowadays however, some of the main interests of EOs are disease control and food preservation. Thus according to several reports, EOs have been proposed as an alternative to synthetic fungicides with promising results (Soliman and Badeaa, 2002; Santamarina et al., 2017; Verdeguer et al., 2020; Giménez-Santamarina et al., 2022).

The Mediterranean basin is especially rich in species of aromatic plants and is a center of their diversification. Species of the genus *Thymus* (*Lamiaceae*) are widely distributed in the Iberian Peninsula, and they form a taxonomically complex group of aromatic species. These plants are traditionally known for their medicinal and culinary purposes, and stand out for their antiseptic, antispasmodic and antitussive medicinal properties (Pina-Vaz et al., 2004; Martínez Marciales et al., 2023). Different *Thymus* species are used mainly as flavorings for foods, such as creams, sauces, vegetables, meat and fish. However, several studies have pointed out their potential as an antimicrobial agent and food preservative for cereals, grains, legumes, fruit and vegetables (Yakoubi et al., 2014; Pandey et al., 2017; Moukhles et al., 2022).

In the last few years, studies about the applications of *Thymus* EOs and their components have grown in number and these EOs been reported as effective against fungal phytopathogens (Santamarina et al., 2017; Costa Junior, 2018; Sapper et al., 2019; Aslam et al., 2022).

Additionally, it has been demonstrated that the biological activity of EOs depends on their chemical composition, which is influenced by factors like plant genotype, geographical origin, and environmental and agronomic conditions (Verdeguer et al., 2020). According to several authors, *Thymus* EOs present a widespread chemical polymorphism (Pina-Vaz et al., 2004; Rota et al., 2008). Thyme EOs are a complex mixture of different components that depend on plant species, of which the main ones are thymol, p-cymene, Ɣ-terpinene, carvacrol, (E)-β-cariophelene and linalool. They possess therapeutic and antimicrobial properties, namely antifungal, antioxidant, anti-inflammatory, etc (Vassiliou et al., 2023).

Currently, abundant information is available on the potential of *T. vulgaris* EOs against phytopathogenic and postharvest fungi, and also against: *Alternaria citri*, the causal agent of black rot in orange; *Fusarium oxysporum* f. sp. *radicis-lycopersici*, *Phytophthora infestans* and *Rhizoctonia solani; Penicillium expansum* and *Penicillium crustosum*, which are associated with the blue mold disease of grapes, among others (Ramezaniana et al., 2016; Ghuffar et al., 2021; Aksit et al., 2022).

Some studies have demonstrated that *T. serpyllum* EOs are able to inhibit the growth of *Verticillium dahliae* during *in vitro* tests (Arslan and Dervis, 2010). Sokolić-Mihalak et al. have revealed the inhibitory *in vitro* effect of *T. serpyllum* EOs against *Aspergillus ochraceus*, *A. carbonarius* and *A. niger* (Sokolić-Mihalak et al., 2012). Interestingly, others authors have proven the potential of the EOs of *T. serpyllum* for use in food preservation due to the dominant presence of thymol and carvacrol (Nedorostova et al., 2009; Galovičová et al., 2021).

Research into the *T. piperella* EO is very scarce and is related to the inhibition of food spoilage by fungi, with some inhibition on *Aspergillus niger* (Ruiz-Navajas et al., 2013).

The ability of EOs to protect foods against not only pathogenic and spoilage microorganisms, but also oxidation, has been reported by several researchers (Alves-Silva et al., 2013; Ruiz-Navajas et al., 2013). To achieve effective antimicrobial activity in direct food applications, high concentrations of EOs are generally needed, which might impact inappropriate flavors and odors in products (Seydim and Sarikus, 2006). To avoid this problem, EOs can be incorporated into bioactive film coatings, which would allow the compound to be fixed and retained on the product surface to, thus, increase its effectiveness. The major compounds in these coatings are biodegradable polymers and a relatively small amount of EOs can be used. Consequently, the application costs of EOs and/or other problems, such as the intense aroma and potential toxicity, could be minimized (Sánchez-González et al., 2010).

The objectives of this work were to: i) determine the chemical composition of the EOs of *Thymus serpyllum* and *Thymus piperella* chemotypes 1 and 2; ii) investigate the antifungal potential of EOs under “*in vitro*” conditions against *Alternaria alternata*, *Bipolaris spicifera*, *Curvularia hawaiiensis*, *Fusarium oxysporum* fsp. *lycopersici*, *Penicillium italicum* and *Botryotinia fuckeliana*; iii) evaluate a natural biofilm with the *T. serpyllum* EO, which we created as an antifungal product study, for rice grain (bomba Valencia Protected Designation of Origin of Valencian Rice), and for cherry tomato fruit conservation.

## 2 Materials and methods

### 2.1 Essential oils

The EOs of *Thymus piperella* chemotypes Tp1 and Tp2 were obtained from wild populations located at La Drova (39° 00’ 12’’ N, 0° 15’ 47’’ W) and La Safor 38°52’19”N, 0°15’ 3” W, both in Spain. The chemical profiles of the EOs from these populations have been previously reported by Boira and Blanquer (1998). Voucher specimens from both populations were kept at the Herbarium of the UPV (Universitat Politècnica de València; VALA no. 9581-9582). The *Thymus serpyllum* EO (CAS Number OF30171), obtained from flowers, was purchased from Pranarom, Avda. Diagonal 472, Barcelona, Spain.

The samples from wild populations (approx. 200 g) were obtained from 50 individual plants in the full flowering stage and were randomly distributed in the sampling area. After removing lignified stems, inflorescences and leaves were air-dried in the dark at room temperature. Samples were divided into three subsamples and weighed about 50 g. They underwent hydrodistillation using a Clevenger-type apparatus for 3 h. After drying with anhydrous sodium sulfate, the EO was diluted to 2% (v/v) in dichloromethane (Sigma-Aldrich, capillary GC grade) and stored in glass vials at -18°C without light until the GC analysis. The Serpyllum EO was diluted and stored in the same way.

### 2.2 Gas chromatography

The samples analysis was performed by gas chromatography with a flame ionization detector (GC-FID) and mass spectrometry (GC-MS). A Clarus 500 GC (Perkin-Elmer Inc. Wellesley. PA. USA) chromatograph, equipped with a FID detector and capillary column ZB-5 (30 m × 0.25 mm i.d. × 0.25 μm film thickness; Phenomenex Inc. Torrance, CA. USA), was used for the quantitative analysis. The injection volume was 1 μL. The GC oven temperature was programmed from 50°C to 250°C at a rate of 3°C min−1. Helium was the carrier gas (1.2 mL min−1). Injector and detector temperatures were set at 250°C. The percentage composition of the EO was calculated from the GC peak areas without correction factors by using the Total Chrom 6.2 software (Perkin-Elmer Inc., Wellesley. PA. USA).

### 2.3 Gas chromatography and mass spectrometry

The GC-MS analysis was carried out on Clarus 500 GC-MS (Perkin-Elmer Inc.) apparatus, equipped with the same capillary column and carrier. Its operating conditions are described above for the GC-FID analysis. The ionization source temperature was set at 200°C and the 70 eV electron impact mode was employed. MS spectra were obtained in the full scan mode (mass range m/z 45-500 uma). The total ion chromatograms (TIC) and mass spectra were processed with the Turbomass 5.4 software (Perkin-Elmer Inc.). Retention indices were determined by an injection of the C_8_–C_25_ n-alkanes standard (Supelco, Bellefonte, PE, USA) under the same conditions. The EO components were identified by making a comparison of the calculated retention indices and high probability matches according to a mass spectra computer library search (NIST MS 2.0) and the available data from the literature (Adams, 2007). The identification of the following compounds was also confirmed by comparing their experimental linear retention index (LRI) to those of authentic reference standards (Merck KGaA, Darmstadt, Germany): α-pinene, β-pinene, camphene, p-cymene, myrcene, limonene, (Z)-β-ocymene, camphor, terpinolene and terpinen-4-ol.

### 2.4 Fungal species

The fungi herein employed were *Alternaria alternata* (AA) CECT 20943, *Curvularia hawaiiensis* (CH) CECT 20934, which were isolated in the Botany Laboratory of the Department of Agroforest Ecosystems (UPV) from the rice samples collected from the “La Albufera” rice-producing Mediterranean Region in Valencia (Spain). The fungal species were morphologically and molecularly identified and then deposited in the Spanish Type Culture Collection (CECT). *Bipolaris spicifera* (BS) CECT 2776 isolated from tobacco, *Fusarium oxysporum* f. sp. *lycopersici* (FOL) CECT 2715 isolated from tomato, *Botryotinia fuckeliana* (BF) CECT 2100 isolated from bean, *Verticillium dahliae* (RS) CECT 2694 isolated from olive, and *Penicillium italicum* (PI) CECT 2294 isolated from orange. They were supplied by the CECT.

### 2.5 Antifungal activity in solid media. Mycelial growth inhibition

The bioassay was performed in Petri dishes (90x15 mm and 150x20 mm) with dissolving 150 and 300 µg/mL (Tween 20, 0.1%) of different Thymus EOs in previously sterilized Potato Dextrose Agar (PDA) growth medium flasks at 45-50°C while the medium was still liquid to be distributed in Petri dishes. Petri dishes were inoculated with an 8 mm-diameter disk of a 7-day old colony on the PDA of each tested fungi. Plates were incubated in the dark at 25°C for 7 days. Fungal growth was evaluated by measuring the colony diameter in two perpendicular directions daily. Six replicate dishes were used for each EO and fungi. The control Petri dishes contained only PDA/Tween 20 (0.1%) and the analyzed fungus.

On day 7, mycelial growth inhibition (MGI) was determined by the following formula (Albuquerque et al., 2006):


MGI =[(DC – DO)/DC] × 100

where DC is the average of the colonies in the control dishes, and DO is the average of the colonies’ diameter in the dishes with oil.

### 2.6 *In vivo* study of the antifungal effect of the *Thymus serpyllum* EO on rice caryopsis. Effect of essential oil on rice storage

Healthy Valencian rice grains were sterilized superficially with sodium hypochlorite (20%) for 5 min, rinsed twice with distilled water and air-dried at room temperature (25 ± 2°C). Then the rice seeds for each tested fungus were dipped into a flask containing 50 mL of a spore suspension of 5 × 105 conidia/mL prepared in water-Tween 20 (0.1%) for 30 min. Finally, they were air-dried to complete dryness.

Then the rice caryopses inoculated with the mold were placed inside 150 × 150 mm^2^ plastic boxes, with 100 seeds per box. Two concentrations (300 and 600 μg/mL) of the *Thymus serpyllum* EO were prepared in Tween 20 (0.1%)/agar 0.25%. Then 5 mL of each solution were sprayed onto boxes. Seeds were wetted with the prepared solutions and dried to complete dryness, and a fine coating formed. The control was prepared similarly to the EOs assay with equal amounts of sterile water/Tween 20 (0.1%), but without the *T. serpyllum* EO. All the boxes were then transferred to storage at 28°C at high relative humidity (90-95% RH) for 20 days. The percentage of infected rice grains was recorded after 15 and 30 days of incubation with an Olympus SZX10 magnifying glass.

### 2.7 *In vivo* antifungal effect of the *Thymus serpyllum* EO on cherry tomato conservation

#### 2.7.1 Preparing the EO solution for fruit coating.

The *T. serpyllum* EO solution for coating fruit was prepared at the 300 and 400 μg/mL concentrations. The EO was homogenized by orbital shaking at 170 rpm for 10 min in flasks containing water/Tween 20 (0.1%)/0.25% agar.

#### 2.7.2 Preparing the fungal inoculum

To recover the fruit with the fungus, a solution containing FOL propagules was prepared. To do so, 10 mL of a suspension of 5x10^5^ conidia/mL of the fungus were added to 90 mL of water/Tween 20 (0.1%)/0.25% agar. The mixture was homogenized by orbital shaking at 170 rpm for 10 min to obtain a homogeneous suspension.

#### 2.7.3 Tomatoes cherry coated with the EO and the fungal inoculum

Cherry tomatoes (origin Mazarrón, province of Murcia, Spain) were sterilized superficially with 1% sodium hypochlorite solution for 2 min and then washed twice with sterile distilled water for 4 min. Fruit were distributed into three batches with 50 fruit each (2 controls and the *T. serpyllum*-film treatment). They were all subjected to a small wound (1 mm depth) on the surface, made with a sterile needle (punch). During the *T. serpyllum*-film treatment, fruit were immersed in the solution containing the T*. serpyllum* EO for 4 min before being placed in racks and dried for 24 h at room temperature. They were then bathed in the fungal inoculum for 2 min. The fruit covered with the FOL suspension were placed in racks. During assay ‘control 1’ (50 fruit), damaged fruit were only bathed with the fungal inoculum. During assay ‘control 2’ (50 fruit), first damaged fruit were immersed for 4 min in the coating solution containing only agar and Tween with no EO before being dried for 24 h and later bathed with the fungal inoculum.

The three lots (control 1, control 2 and the *T. serpyllum*-film treatment) were placed in a chamber at the same time (85% RH at 210°C). Cherry tomato evolution was controlled for 7 and 14 days.

### 2.8 Statistical analysis

The fungal growth results were submitted to an analysis of variance (ANOVA). The HSD Tukey intervals were represented to compare species and treatment, with significant values at P<0.05. The data analysis was performed by the Statgraphics Centurion XVII software (Stat Point, Inc., Herndon, Virginia, USA).

## 3 Results

### 3.1 Chemical composition of EOs

**Table 1** shows the main components and their relative contents of the *T. serpyllum* EOs, as well as the two EOs of *T. piperella* chemotypes 1 and 2 (Tp1 and Tp2).

**Table 1:** Chemical composition of the *Thymus serpyllum*, *Thymus piperella* TP1 and *Thymus piperella* Tp2 essential oils.

Compounds^1^	LRI^2^	LRI (lit.)^3^	*T. serpyllum*	*T. piperella* Tp1	*T. piperella* Tp2
			%	%	%
(2E)-hexenal	848	846	0.2	0.1	tr^4^
Tricyclene	922	921	tr	0.1	tr
a-Thujene	926	924	0.3	0.2	1.7
a-Pinene	933	932	0.9	0.7	0.9
Camphene	949	946	0.9	1.7	0.3
Sabineno	973	969	- ^5^	–	0.1
b-Pinene	978	974	0.2	0.2	0.2
1-octen-3-ol	980	974	1.1	–	–
octan-3-one	980	979	tr	0.3	0.3
Myrcene	989	988	0.8	0.2	2.1
a-Phellandrene	1007	1002	0.1	tr	0.2
d-3-carene	1009	1008	tr	tr	0.1
a-Terpinene	1016	1014	0.5	0.1	2.6
p-cymene	1026	1020	12.3	8.7	22.7
Limonene	1029	1024	0.7	0.4	0.4
1,8-Cineole	1032	1026	1.3	0.9	0.1
Cis-b-Ocimene	1038	1032	tr	tr	–
trans-b-Ocimene	1048	1044	–	tr	0.1
g-Terpinene	1060	1054	3.7	1.5	22.2
Sabinene hydrate<cis>	1070	1065	0.1	0.6	–
Linalool oxide<cis-> furanoid	1071	1067	–	0.3	0.6
Mentha-2,4(8)-diene<ρ->	1085	1085	tr	–	–
Camphenilone	1086	1078	tr	–	0.1
Linalool oxide<trans->	1087	1084	–	tr	0.1
trans-Sabinen hydrate	1098	1098	–	tr	–
Linalool	1101	1095	6.1	4.1	1.3
Menth-2-en-1-ol<cis-p>	1122	1118	–	tr	–
a-Campholenal	1125	1122	–	0.1	–
Camphor	1147	1141	1.2	11.9	–
Pinocarvone	1161	1160	–	tr	–
Borneol	1173	1165	2.5	4.6	0.4
Terpinen-4-ol	1181	1174	1.7	1.4	0.6
p-Cymen-8-ol	1188	1179	–	0.6	–
a-Terpineol	1195	1186	1.5	0.5	–
cis-Dihydrocarvone	1196	1191	tr	tr	tr
trans-Dihydrocarvone	1204	1200	–	0.1	–
Cis-Ocimenone	1226	1226	0.1	–	–
Isobornyl formiate	1227	1235	–	tr	–
Thymol methyl ether	1229	1232	0.2	–	–
Cuminaldehyde	1238	1238	–	0.1	–
*Trans*-Ocimenone	1239	1235	–	–	1.9
Carvacrol methyl ether	1243	1241	0.4	0.1	–
Carvone	1245	1239	0.3	tr	–
Geraniol	1254	1249	12.4	–	–
Bornyl acetate	1284	1284	0.5	0.7	0.1
Thymol	1297	1289	21.5	1.8	35.7
Carvacrol	1305	1298	18.7	51.0	1.0
a-Cubebene	1348	1348	0.2	tr	–
Eugenol	1352	1356	0.2	–	–
Linalool isobutanoate	1358	1373	tr	–	–
Thymol acetate	1364	1349	–	0.3	0.1
a-Copaene	1375	1374	0.1	–	–
Geranyl acetate	1379	1379	4.4	–	–
b-Bourbonene	1382	1387	–	0.2	–
b-Elemene	1393	1389	–	tr	–
Methyl eugenol	1400	1403	0.1	–	–
b-Caryophyllene	1419	1417	2.2	2.4	3.2
b-Copaene	1430	1430	–	tr	–
Aromadendrene	1437	1439	0.1	tr	0.1
alpha-Humulene	1454	1452	0.8	0.1	0.1
Aromadendrene allo	1459	1458	0.1	tr	–
Germacrene D	1478	1484	0.1	0.1	0.1
g-muurolene	1480	1478	–	tr	–
b-selinene	1490	1489	–	–	tr
Bicyclogermacrene	1494	1500	0.1	0.1	0.1
b-Bisabolene	1506	1505	0.1	–	0.1
g-Cadinene	1512	1513	–	0.1	tr
Geranyl isobutanoate	1517	1514	0.3	–	–
d-Cadinene	1517	1522	–	tr	0.1
Elemol	1548	1548	tr	–	tr
Spathulenol	1576	1577	0.5	0.1	0.1
Caryophyllene oxide	1581	1582	–	3.0	0.2
Viridiflorol	1599	1592	–	tr	–
Humulene epoxide (II)	1609	1608	0.1	0.1	–
a-Acorenol	1638	1632	0.3	tr	–
b-Eudesmol	1655	1649	–	tr	0.1
a-cadinol	1673	1652	–	tr	–
**Hydrocarbon monoterpenes**			**21.7**	**14.7**	**53.6**
**Oxygenated monoterpenes**			**72.0**	**78.1**	**41.8**
**Hydrocarbon sesquiterpenes**			**3.6**	**2.9**	**3.8**
**Oxygenated sesquiterpenes**			**0.9**	**3.1**	**0.4**
**Other compounds**			**1.2**	**0.4**	**0.3**
**Total identified**			**99.37**	**99.19**	**99.83**

^1^Compounds listed according to their elution order in a DB-5 column.

^2^LRI values.

^3^LRI values from the literature (Adams, 2007).

^4^tr: traces (% < 0.05).

^5^Not detected.

Bold values are the generic value of the chemical group.

Fifty-two compounds were identified in the *T. serpyllum* EO, which accounted for 99.37% of composition. The major compounds were thymol (21.5%) and carvacrol (18.7%). In the *T. piperella* tp1 EO, 60 compounds were identified and corresponded to 99.19% of composition, where carvacrol was the main compound. EO *T. piperella* tp2 contained 42 compounds (99.83%) with thymol (35.7%) as the most prevalent component. Along with phenolic compounds thymol and carvacrol, their metabolic precursors p-cymene and γ-terpinene also showed noticeable proportions, especially in the *T. piperella* oil from La Safor.

As chemical profiles can be defined based on the composition of the dominant compound, we defined the following chemical profiles according to our results: thymol/carvacrol for *T. serpyllum*, carvacrol for *T. piperella* Tp1 and thymol for *T. piperella* Tp2. Monoterpenes (hydrocarbon and oxygenated monoterpenes) were the most abundant compounds of the EOs of *T. serpyllum*, *T. piperella* Tp1 and *T. piperella* Tp2 analyzed with a total of 93.7%, 92.8% and 95.4% of EO, respectively. The oxygenated monoterpenes were predominant in *T. serpyllum* and *T. piperella* Tp1, while those in hydrocarbon-oxygenated *T. piperella* Tp2 were slightly higher than the oxygenated monoterpenes. When comparing the three EOs profiles, it is worth highlighting the balanced proportion of thymol and carvacrol in the *T. serpyllum* oil, while all these compounds predominated in the *T. piperella* chemotypes.

### 3.2 *In vitro* studies. Determining the antifungal potential of EOs. MGI (%) (mycelial growth inhibition)

The results obtained in this assay showed that the EOs of *T. serpyllum* and the two of *T. piperella* (Tp1 and Tp2) reduced the fungal growth of all the evaluated phytopathogens (**Tables 2**–**4**). The inhibition of EOs was affected for the doses used in all the tested fungi. The MGI increased the higher doses became. *C. hawaiiensis*, FOL and B. spicifera were the most sensitive fungi to the three evaluated EOs. We highlight a significant reduction in *T. piperella* Tp2 against *A. alternata* at 300 µg/mL.

**Table 2:** Effects of the *Thymus serpyllum* essential oil at 150 and 300 µg/mL on colonies diameter growth and MGI (Mycelial Growth Inhibition) of *Alternaria alternata* (AA), *Bipolaris spicifera* (BS), *Curvularia hawaiiensis* (CH), *Fusarium oxysporum lycopersici* (FOL), *Penicillium italicum* (PI) and *Botryotinia fuckeliana* (BF).

TREATMENT	AA	BS	CH	FOL	PI	BF
**Control**	64.10	81.80	43.10	67.60	22.50	60.40
**150 µg/mL**	35.50	13.20	3.00	20.00	15.90	26.30
**300 µg/mL**	28.40	7.30	0.00	2.60	4.60	14.50
MGI	AA	BS	CH	FOL	PI	BF
**150 µg/mL**	44.62	83.86	93.04	70.41	29.33	56.46
**300 µg/mL**	55.69	91.08	100	96.15	79.56	75.99

**Table 3:** Effects of the *Thymus piperella* Tp1 essential oil at 150 and 300 µg/mL on colonies diameter growth and MGI (Mycelial Growth Inhibition) of *Alternaria alternata* (AA), *Bipolaris spicifera* (BS), *Curvularia hawaiiensis* (CH), *Fusarium oxysporum lycopersici* (FOL), *Penicillium italicum* (PI) and *Botryotinia fuckeliana* (BF).

TREATMENT	AA	BS	CH	FOL	PI	BF
**Control**	64.10	81.80	43.10	67.60	22.50	60.40
**150 µg/mL**	57.50	47.40	35.50	52.00	21.20	49.70
**300 µg/mL**	34.00	7.50	20.80	14.00	17.40	17.30
MGI	AA	BS	CH	FOL	PI	BF
**150 µg/mL**	10.30	42.05	17.63	23.08	5.78	17.72
**300 µg/mL**	46.96	90.83	51.74	79.29	21.78	71.36

**Table 4:** Effects of the *Thymus piperella* Tp2 essential oil at 150 and 300 µg/mL on colonies diameter growth and MGI (Mycelial Growth Inhibition) of *Alternaria alternata* (AA), *Bipolaris spicifera* (BS), *Curvularia hawaiiensis* (CH), *Fusarium oxysporum lycopersici* (FOL), *Penicillium italicum* (PI) and *Botryotinia fuckeliana* (BF).

TREATMENT	AA	BS	CH	FOL	PI	BF
**Control**	64.10	81.80	43.10	67.60	22.50	60.40
**150 µg/mL**	33.90	32.40	29.10	20.10	18.90	51.20
**300 µg/mL**	9.60	6.60	2.20	7.30	16.80	17.60
MGI	AA	BS	CH	FOL	PI	BF
**150 µg/mL**	47.11	60.39	32.48	70.27	16.00	15.23
**300 µg/mL**	85.02	91.93	94.90	89.20	25.33	70.86

The *T. serpyllum* EO (thymol/carvacrol chemotype) showed strong inhibition (100% to 93% MGI) against *C. hawaiiensis*. It totally inhibited fungus growth at 300 µg/mL and slightly decreased it at 150 µg/mL. However, this oil also showed strong inhibition against FOL and *B. spicifera* at 300 µg/mL. In the latter, it displayed marked inhibition at 150 µg/mL. The other obtained MGI values ranked between 80% and 50%.

The *T. piperella* EO of Tp1 (carvacrol chemotype) obtained a high MGI value at 300 µg/mL against *B. spicifera* (90.83%), *F. oxysporum lycopersici* (79.29%) and *B. fuckeliana* (71.36%). The other fungi showed minor inhibition with MGI values of ≤ 51.74% (**Table 3**).

Major MGI values were obtained for the *T. piperella* EO (Tp2) (thymol chemotype) against *C. hawaiiensis*, *B. spicifera*, FOL and *A. alternata* at 300 µg/mL, with very high MGI values of 94.90%, 91.93%, 89.20% and 85.02%, respectively, and lower values against *B. fuckeliana* with MGI values of 70.86% (**Table 4**). At 150 µg/mL, this oil also obtained good MGI values against FOL and *B. spicifera* (70.27% and 60.39%, respectively), and lower values against other fungi.

**Figure 1:**
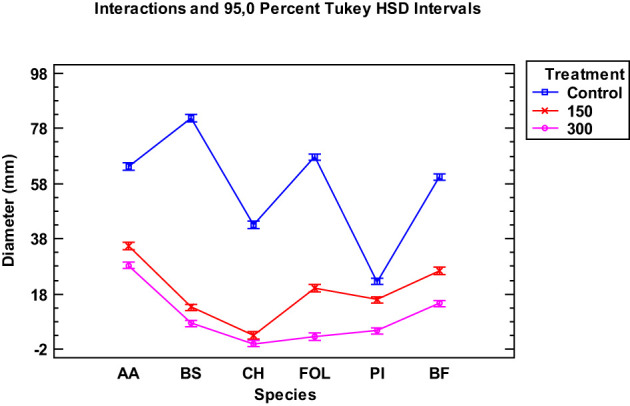
Interaction plot, mean growth, species, 150 and 300 μg/mL concentrations of the *Thymus serpyllum* essential oils against *Alternaria alternata* (AA), *Bipolaris specifera* (BS), *Curvularia hawaiiensis* (CH), *Fusarium oxysporum lycopersici* (FO), *Penicillium italicum* (PI) and *Botryotinia fuckeliana* (BF). n (30) observations per treatment were used in the statistical analysis.

The inhibitory effect of the *Thymus serpyllum* EO on the growth of six fungal species at 150 and 300 µg/mL was evaluated by Tukey’s HSD plots. The results showed a significant MGI for all the species tested at both doses compared to the control (p<0.05) (**Figure 1**). This inhibition was greater at the 300 µg/mL dose, as the graphs depict. When comparing the two tested doses, the difference between the mycelial growth of the fungal species was significant, except for *Curvularia hawaiiensis*.

**Figure 2:**
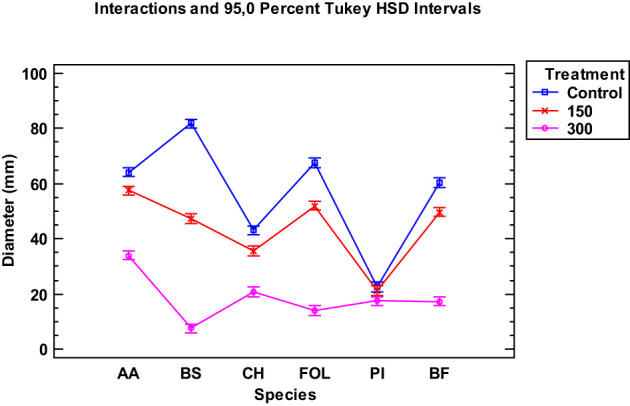
Interaction plot, mean growth, species, 150 and 300 μg/mL concentrations of the *Thymus piperella* Tp1 essential oils against *Alternaria alternata* (AA), *Bipolaris specifera* (BS), *Curvularia hawaiiensis* (CH), *Fusarium oxysporum lycopersici* (FO), *Penicillium italicum* (PI) and *Botryotinia fuckeliana* (BF). n (30) observations per treatment were used in the statistical analysis.

**Figure 3:**
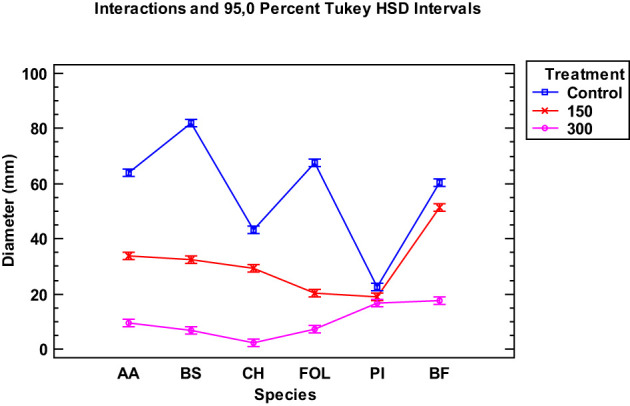
Interaction plot, mean growth, species, 150 and 300 μg/mL concentrations of the *hymus piperella* Tp2 essential oils against *Alternaria alternata* (AA), *Bipolaris specifera* (BS), *Curvularia hawaiiensis* (CH), *Fusarium oxysporum lycopersici* (FO), *Penicillium italicum* (PI) and *Botryotinia fuckeliana* (BF). n (30) observations per treatment were used in the statistical analysis.

When examining the data collected from the two EOs of the tested *Thymus piperella* cultivars, only for the *Penicillium italicum* species were the extracts of cultivars not as effective in inhibiting the fungus as in *Thymus serpyllum*. There was no statistically significant difference between the control and the *Thymus piperella* Tp1 EO at 150 µg/mL (**Figures 2**, **3**).

**Figure 4:**
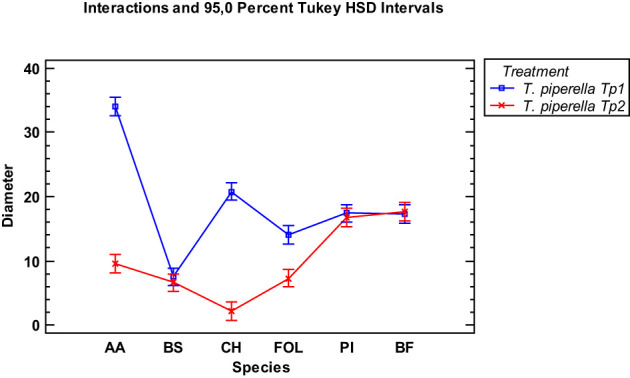
Interaction plot, mean growth, species, 300 μg/mL concentrations of the *Thymus piperella* Tp1 and *Thymus piperella* Tp2 essential oils against *Alternaria alternata* (AA), *Bipolaris specifera* (BS), *Curvularia hawaiiensis* (CH), *Fusarium oxysporum lycopersici* (FO), *Penicillium italicum* (PI) and *Botryotinia fuckeliana* (BF). n (30) observations per treatment were used in the statistical analysis.

When the essential oils of the two *Thymus piperella* cultivars were compared at the 300 µg/mL dose, cultivar Tp2 was more effective in inhibiting the *Alternaria alternata*, *Curvularia hawaiiensis* and *Fusarium oxysporum* f.sp. *lycopersici* species. Inhibition on the other fungi was similar (**Figure 4**).

**Figure 5:**
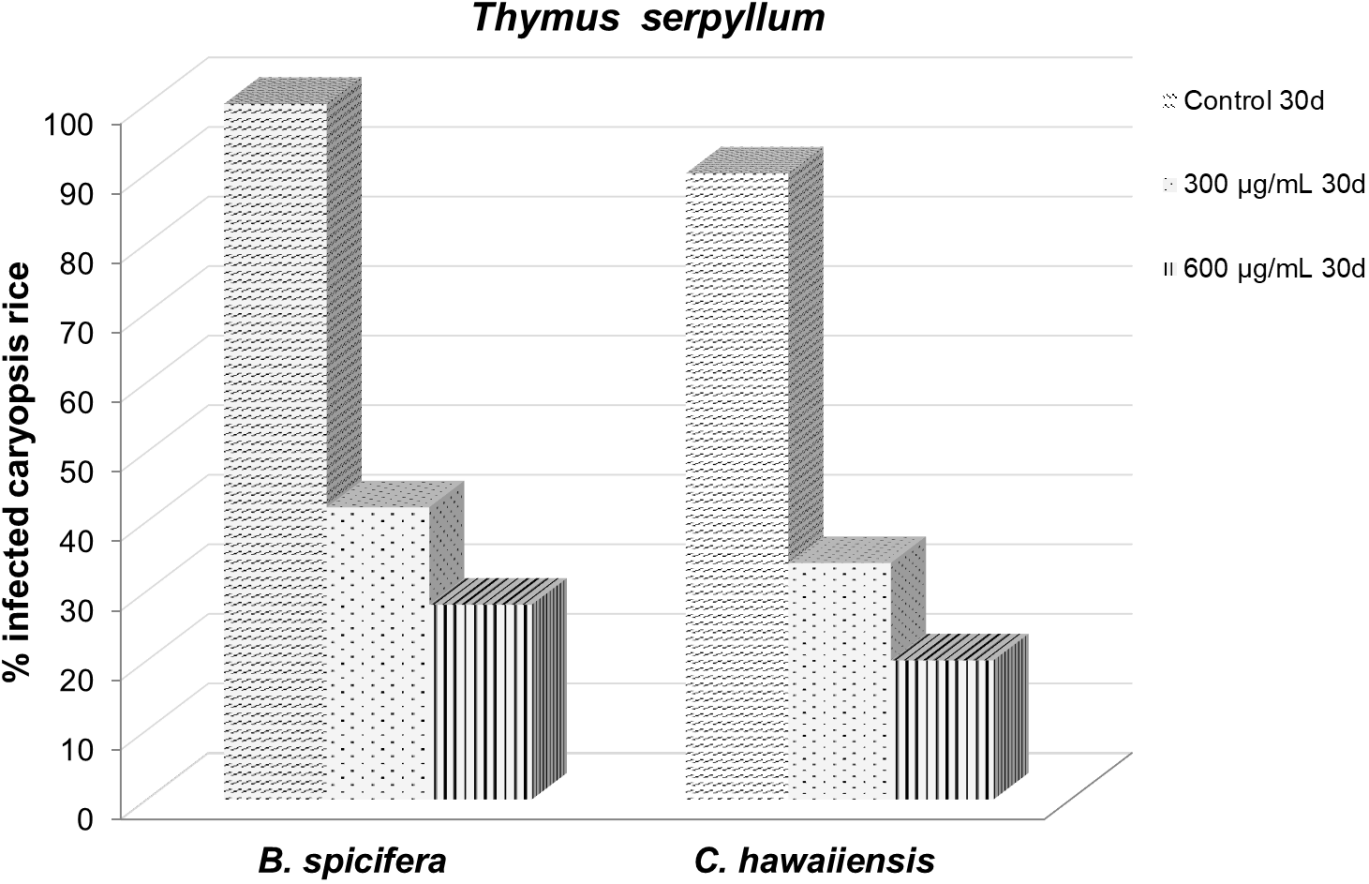
Effect of *Thymus serpyllum* essential oil at different concentrations (300 and 600 µg/mL) on *Bipolaris spicifera* (BS) and *Curvularia hawaiiensis* (CH) fungi inoculated in rice grains for 30 days.

Finally, the results showed that, when comparing the inhibitory effect of the three EOs tested at the 300 µg/mL concentration, the *Thymus serpyllum* extract had higher inhibitory capacity on the growth of *Bipolaris spicifera, Curvularia hawaiiensis* and *Fusarium oxysporum* f.sp. *lycopersici* (**Figure 5**). At this concentration (300 μg/mL), the *T. serpyllum* EO exerted superior antifungal activity than the other tested EOs. Therefore, the *Thymus serpyllum* EO was selected to study its effect on harvested and stored rice conservation, and also on cherry tomato conservation, to extend their commercial shelf lives.

### 3.3 *In vivo* study of the antifungal effect of the *Thymus serpyllum* EO on rice caryopsis. Essential oil on rice storage

The protective effect of the film created at the 300 and 600 µg/mL doses against the fungi *Bipolaris spicifera* and *Curvularia hawaiiensis*, which attacked rice during storage, was similar on the studied fungi, with no significant differences between them at 15 days (**Table 5**; **Figure 5**). However after 30 trial days, the 600 µg/mL dose was much more effective, with an infection rate of 20% and 28% against the fungi CH and BS, respectively.

**Table 5:** Effect of *Thymus serpyllum* essential oil at different concentrations (300 and 600 µg/mL) on *Bipolaris spicifera* (BS) and *Curvularia hawaiiensis* (CH) fungi inoculated in rice grains for 15 and 30 days.

Species	Treatment	15 days		30 days	
		spoiled (%)	healthy (%)	spoiled (%)	healthy (%)
	Control	80	20	100 a	0
BS	Ts-300 µg/mL	30	70	42 b	58
	Ts-600 µg/mL	23	77	28 c	72
	Control	70	30	90 a	10
CH	Ts-300 µg/mL	22	78	34 b	66
	Ts-600 µg/mL	14	86	20 c	80

Control: Rice caryopsis without film and no EO.

Ts-300 µg/mL: Rice caryopsis with film and EO at 300 µg/mL.

Ts-600 µg/mL: Rice caryopsis with film and EO at 600 µg/mL.

Different letters in the same column indicate a significant difference at 95% level probability by Tukey’s HSD.

Different letters in the same column indicate a significant difference at 95% level probability by Tukey’s HSD.

### 3.4 *In vivo* antifungal effect of the *Thymus serpyllum* EO on cherry tomato conservation

In this study, the protective effect of the film without EO was observed because it maintained fruit turgor, prevented their weight loss, allowed FOL infection to advance and functioned as a second epidermis.

**Figure 6:**
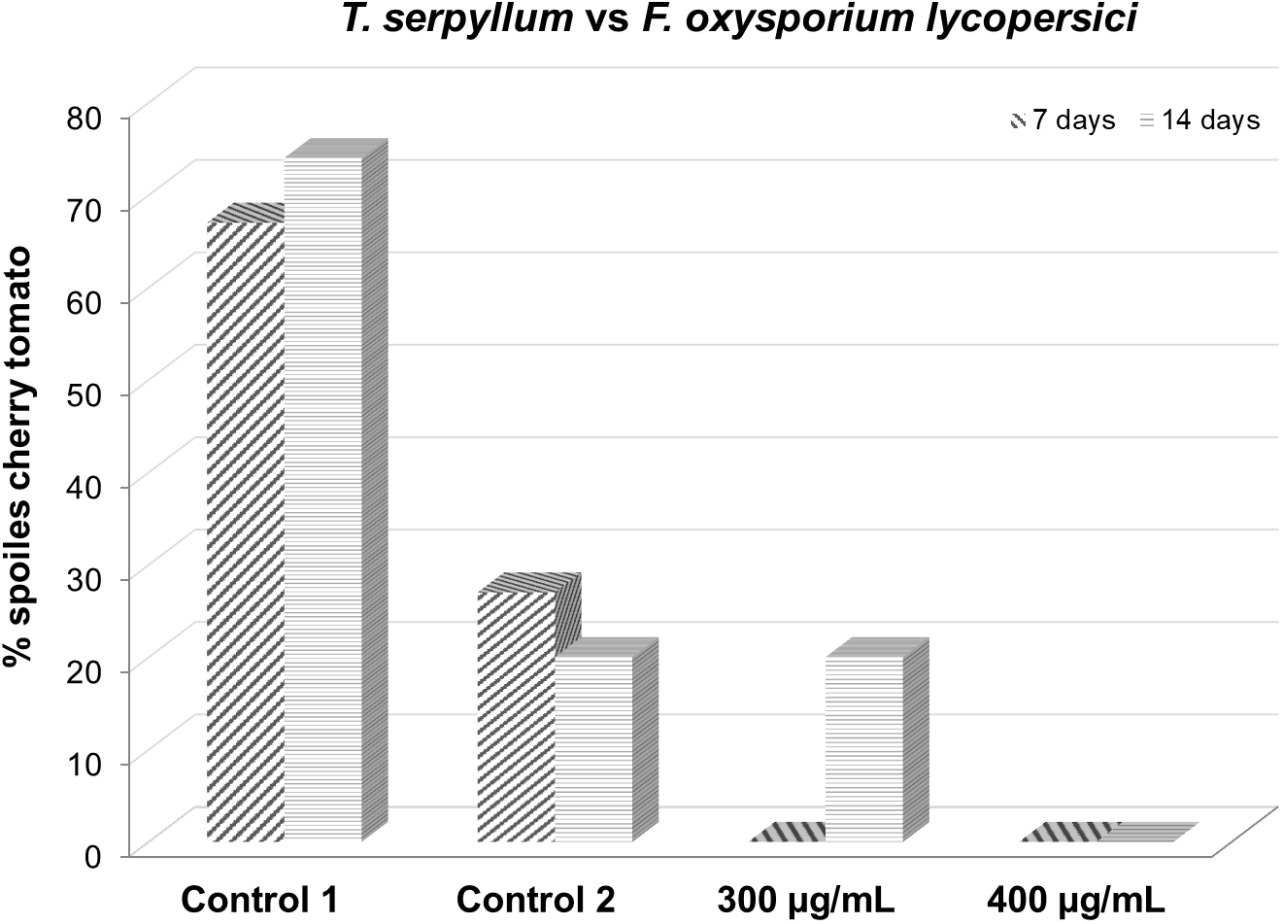
Effect of *Thymus serpyllum* essential oil at different concentrations (300 and 400 µg/mL) on *Fusarium oxysporum lycopersici* inoculated in cherry tomato for 7 and 14 days.

After 7 days, both the 300 and 400 µg/mL doses were equally effective, with total protection of 100% healthy fruits. Over time, the 400 µg/mL dose remained effective for up to 14 testing days. Starting on day 14, the protective effect of the 300 µg/mL dose began to decrease (**Table 6**; **Figure 6**).

**Table 6:** Effect of *Thymus serpyllum* essential oil at different concentrations (300 and 400 µg/mL) on *Fusarium oxysporum lycopersici* inoculated in cherry tomato for 7 and 14 days.

Species	Treatment	7 days		14 days	
		spoiled (%)	healthy (%)	spoiled (%)	healthy (%)
	Control 1	67	33	74 a	26
FOL	Control 2	27	73	34 b	66
	Ts-300 µg/mL	0	100	20 c	80
	Ts-400 µg/mL	0	100	0 d	100

Control 1: Cherry tomato without film and no EO.

Control 2: Cherry tomato with film and no EO.

Ts-300 µg/mL: Cherry tomato with film and EO at 300 µg/mL.

Ts-400 µg/mL: Cherry tomato with film and EO at 400 µg/mL.

Different letters in the same column indicate a significant difference at 95% level probability by Tukey’s HSD.

The 600 µg/mL dose was not used for this trial because it confers fruit flavor and damages its cuticle. It could be used with rice because this cereal must be husked to be eaten.

## 4 Discussion

EOs are widely known for their several biological activities, such as bactericides, fungicides, insecticides, and also for their culinary and medicinal uses. Hence they have applications in food and pharmaceutical industries (Plaza et al., 2004; Jahani et al., 2020; Raveau et al., 2020). Of *Lamiaceae* family members, the genus *Thymus* comprises around 350 species and 36 subspecies with wild species and cultivated plants, and the Iberian Peninsula in general, and the Mediterranean Basin in particular, are centers of diversification. Most indigenous species grow all around the Mediterranean region (Zeljković and Maksimović, 2015; Vassiliou et al., 2023). The *Thymus* EOs are widely known for their high content of bioactive compounds, such as phenolic acids, flavonoids and terpenes, and for the diversity of chemotypes (Ruiz-Navajas et al., 2013; Yakoubi et al., 2014; Pandey et al., 2017; Moukhles et al., 2022). According to previous studies, their properties depend on their biologically composition (Vassiliou et al., 2023).

Thymol and carvacrol are two phenolic monoterpenes cited as the main components of the EOs of some *Laminaceae* and are produced by aromatic plants as a chemical defense mechanism upon exposure to biotic (herbivores, pathogens, pests) and abiotic factors as environmental stresses (Pulido et al., 2012; Naghdi Badi et al., 2017). Zeljović and Maksimović investigated 45 *Thymus* taxa from the Balkan Peninsula for their composition, where the aromatic thymol, carvacrol and p-cymene chemotypes were the most abundant (Zeljković and Maksimović, 2015).

The *T. vulgaris* EO is the most studied in the species belonging to the genus *Thymus*. These oils are rich in thymol and carvacrol, and are known to be effective against the fungi that infect humans, and also against phytopathogens (Affesa et al., 2022). According to several authors, high thymol and carvacrol concentrations are responsible for antimicrobial and antifungal properties (Yeddes et al., 2018; Kovačević et al., 2021).

Preliminary reports have demonstrated the antifungal activity of the EOs obtained from the plant species belonging to the genus *Thymus* against phytopathogens, such as *P. expansum*, *Botrytis cinerea*, *Monilinia fructicola* and *Rhizopus oryzae*, and cause postharvest fruit diseases of which thymol and carvacrol are their major components (Venturini et al., 2002; Plaza et al., 2004). Arora et al. reported that the *T. vulgaris* EOs showed 100% MGI against the fungi *Colletotrichum capsici*, *Pythium aphanidermatum* and *Fusarium oxysporum*, which cause significant pepper fruit *Capsicum anuum* losses (Arora et al., 2023).

The effect of the *T. zygis* EO has been demonstrated as a preservative in stored products by reducing postharvest fungi development and extending products’ commercial shelf lives (Sapper et al., 2019). Applying EOs to food as coatings helps to maintain the quality of products of natural origins, such as fruit and vegetables, by prolonging their shelf lives, and also with products that are environmentally friendly and safe for humans (Verdeguer et al., 2020).

Our study found that the major components of the (commercial) *T. serpyllum* EO were thymol (21.5%) and carvacrol (18.5%), followed by geraniol (12.4%) and p-cymene (12.3%), of 52 compounds. We demonstrated the fungistatic and fungicidal potentials of these EOs against *A. alternata*, *B. spicifera*, *C. hawaiiensis*, FOL, *P. italicum* and *B. fuckeliana*. Particularly, *C. hawaiiensis* was totally inhibited (100%), as was FOL (96%) at higher doses (300 µl/mL). Thus we highlight the effect of this EO against all the pathogens evaluated according to the obtained MGI values. Interestingly, several authors attribute their properties for both the pharmaceutical industry and food conservation purposes to thymol and carvacrol contents (Sokolić-Mihalak et al., 2012; Naghdi Badi et al., 2017).

Our results agree with preliminary reports, albeit with some variation in the reported percentages, that the major compounds of the *T. serpyllum* EO from plants that grow in the Slovak region were thymol (18.8%), carvacrol (17.4%), o-cymene (15.4%) and geraniol (10.7%) (Galovičová et al., 2021). According to their antimicrobial and antifungal studies, these authors suggested that the *T. serpyllum* EO could be used for storing root vegetables, and also as a *Penicillium* inhibitor in bread. Other studies have revealed that the dominant component of the *T. serpyllum* EO extracted from plants harvested in the flowering stage in Turkey were p-cymene, thymol and Ɣ-terpinene, which has a potential antifungal effect against *Verticillium dahliae* (Arslan and Dervis, 2010). Additionally, the *Thymus serpyllum* EO has a strong inhibitory effect against the growth and mycotoxin production of *Aspergillus ochraceus*, *A. carbonarius* and *A. niger*, which spoil food products and produce ochratoxin A (OTA), an important mycotoxin (Sokolić-Mihalak et al., 2012). These authors noted a significant inhibitory effect at lower doses (100 μL mL^-1^) than those we used (150 and 300 μg mL^-1^). However, we highlight in their tests that the EO was dissolved in 96% ethanol, which alone has a fungicidal effect.

Very few studies have been carried out with *T. piperella*. The few works that exist focused on studies related mainly to food conservation to reduce the oxidation and microbiological degradation caused by bacterial and fungi, which produce rancid odors and flavors, and come with widely known human and animal health concerns (Ruiz-Navajas et al., 2012; 2013). These authors reported the inhibition of fungal foodborne *A. alternata*, *A. niger*, *A. flavus*, *P. chrysogenum*, *Mucor racemosus* and *M. circinelloides*.

In our work, the *T. piperella* EOs were obtained from plants growing in two different locations in the Valencia region of Spain in the flowering stage. According to our results, we were able to determine two chemotypes. To Tp1, we assigned the chemotype carvacrol and chemotype thymol to Tp2. Our results agree with other authors, who have demonstrated that the major component of the *T. piperella* EO were monoterpenes, with phenolic compounds like thymol (Llorens-Molina et al., 2022). So previous studies about the chemical composition of 31 populations of *T. piperella* have demonstrated that an intraspecific variation of EOs compositions can determine three chemotypes according to major components: chemotype A p-cymene-carvacrol-Ɣ-terpinene; chemotype B: p-cymene-thymol, and chemotype C: p-cymene-carvacrol (Llorens-Molina et al., 2022). Several studies have proven the intraspecific variability of *T. piperella* populations, which determine different chemotypes (Boira and Blanquer, 1998; Llorens-Molina et al., 2022).

In our work, the major component of EOs were thymol and carvacrol, with a high potential against the five evaluated phytopathogens. All the studied fungi were more sensitive to *Thymus serpyllum*, whose main components are thymol and carvacrol. Therefore, we suggest that it may be related to the synergistic effects of its components. Both *T. serpyllum* and *T. piperella* had excellent results against *Curvularia hawaiiensis*, *Bipolaris spicifera* and *Fusarium oxysporum*. *T. serpyllum* grows in central and northern Europe, while *T. piperella* grows essentially in the Mediterranean basin. In addition, these EOs could be incorporated into bioactive films by retaining active compounds and to also increase their antifungal potential.

## 5 Conclusions

In this work, the EOs of *T. serpyllum* and the two of *T. piperella* (Tp1 and Tp2) reduced the fungal growth of all the evaluated phytopathogens. However, we emphasize the potential of the *T. serpyllum* EO because all the fungal pathogens were very sensitive, which markedly reduced growth, and even totally against *C. hawaiiensis*, but also with very good results against *Bipolaris spicifera* and *Fusarium oxysporum*. The natural biofilm created from *Thymus serpyllum* proved extremely effective in rice storage compared to the losses caused by the fungi *Curvularia hawaiiensis* and *Bipolaris spicifera*. It is also capable of controlling the *Fusarium oxysporum* fungus in cherry tomatoes during the postharvest and, thus, prolongs their shelf life in both warehouses and stores.

Considering our results and the fact that EOs have different advantages *versus* synthetic fungicides, for instance, they are not toxic to humans and animals, and they are biodegradable with short lives, we suggest that these natural products formulated as biofilms could be safely applied for food conversation purposes to replace synthetic products.

A natural film was obtained at a low *Thymus serpyllum* concentration that is not harmful for human health or the environment.

## Data availability statement

The raw data supporting the conclusions of this article will be made available by the authors, without undue reservation.

## Author contributions

JR: Conceptualization, Data curation, Investigation, Methodology, Resources, Writing – review & editing. FS-F: Data curation, Formal analysis, Investigation, Writing – review & editing. JL-M: Data curation, Formal analysis, Investigation, Methodology, Resources, Writing – review & editing. SL: Writing – review & editing. MPS: Conceptualization, Funding acquisition, Methodology, Project administration, Supervision, Writing – review & editing.
